# Differential Impact of the Supportive Services for Veteran Families (SSVF) Program on Veterans Affairs Healthcare Utilization and Costs Among Rural and Urban Veterans Experiencing Housing Instability

**DOI:** 10.1111/jrh.70167

**Published:** 2026-05-19

**Authors:** Minkyoung Yoo, Alec B. Chapman, Ying Suo, Thomas Byrne, Ann E. Montgomery, Richard E. Nelson

**Affiliations:** ^1^ US Department of Veterans Affairs IDEAS Center, Salt Lake City Health Care System Salt Lake City Utah USA; ^2^ Department of Internal Medicine University of Utah School of Medicine Salt Lake City Utah USA; ^3^ Department of Population Health Sciences University of Utah School of Medicine Salt Lake City Utah USA; ^4^ Center for Healthcare Organization and Implementation Research VA Bedford Healthcare System Bedford Massachusetts USA; ^5^ Boston College School of Social Work Chestnut Hill, Newton, Massachusetts USA; ^6^ National Center on Homelessness among Veterans VA Homeless Programs Office Washington District of Columbia USA; ^7^ US Department of Veterans Affairs Birmingham VA Health Care System Birmingham Alabama USA; ^8^ School of Public Health University of Alabama at Birmingham Birmingham Alabama USA

**Keywords:** healthcare costs, healthcare utilization, housing instability, rural–urban difference, Veterans

## Abstract

**Purpose:**

This study examined whether the Supportive Services for Veteran Families (SSVF) program has a differential impact on healthcare utilization and costs among rural and urban Veterans experiencing housing instability. Understanding geographic variation in program effects is important for guiding implementation and resource allocation within the Veterans Affairs (VA) healthcare system.

**Methods:**

We conducted a retrospective cohort study using national VA administrative data from October 1, 2018 to September 30, 2023. Veterans were classified as SSVF participants or eligible non‐SSVF Veterans based on indicators of housing instability. A target trial emulation framework with inverse probability weighting was used to adjust for baseline differences. Rurality was defined using Rural–Urban Commuting Area codes applied to Veterans’ residential addresses recorded in VA administrative data. Weighted longitudinal models estimated quarterly changes in healthcare utilization and costs and tested whether effects varied by rurality.

**Findings:**

SSVF enrollment was associated with reductions in inpatient utilization and costs and modest increases in outpatient visits, resulting in overall decreases in total VA healthcare costs. Emergency department use showed small reductions among urban Veterans and little measurable change among rural Veterans. Overall patterns of healthcare utilization and spending were similar across rural and urban Veterans, and statistical tests did not indicate significant rural–urban differences in SSVF effects.

**Conclusions:**

SSVF participation was associated with shifts away from inpatient care and toward greater outpatient engagement among Veterans experiencing housing instability. These patterns were observed among both rural and urban Veterans, suggesting that the healthcare benefits of housing stabilization programs are similar across geographic settings.

## 1 Introduction

Rural Veterans experience disparities in healthcare access, utilization, and outcomes compared to urban Veterans [1, 2, 3]. Geographic isolation and long travel distances increase the physical burden of accessing care, whereas limited provider availability and infrastructure constraints, such as fewer healthcare facilities and specialty services, limit the availability of local care and delay access to specialty services [4]. Transportation barriers can further delay or disrupt appropriate care [3, 5]. These challenges are particularly critical for Veterans experiencing housing instability, who often rely on coordinated healthcare and housing support services [6, 7]. When outpatient care is delayed or interrupted, Veterans may turn to the emergency department or require hospitalization for conditions that could have been managed earlier, often resulting in increased costs and poorer health over time [8, 9].

The US Department of Veterans Affairs (VA) provides several programs to prevent and address homelessness among Veterans. One such program, the Supportive Services for Veteran Families (SSVF) program, focuses on rapid re‐housing and homelessness prevention. These services are delivered through community‐based grantees and include temporary financial assistance, case management, and service coordination [10]. Prior studies have shown that SSVF participation is associated with improved housing stability [11, 12, 13, 14] and reduced emergency department and inpatient utilization, along with modest increases in outpatient visits, suggesting a shift toward more preventive care [15, 16]. However, little is known about whether these effects differ across rural and urban settings, where patterns of healthcare access and utilization may differ substantially.

Whether the benefits of SSVF translate similarly for rural and urban Veterans remains unclear. Differences in healthcare access and service infrastructure across geographic settings may influence how housing interventions affect healthcare utilization and costs. Examining whether SSVF influences healthcare utilization and costs differently for rural versus urban Veterans can help clarify how this nationwide VA program reaches Veterans with housing instability across both rural and urban communities. This information is important for evaluating the extent to which SSVF benefits rural Veterans and for informing strategies to ensure the program effectively supports them given the unique challenges they face compared with urban Veterans.

This study addresses this gap by estimating the impact of SSVF enrollment on VA inpatient, outpatient, and emergency department utilization and costs among Veterans experiencing housing instability, and by assessing whether these effects differ by rurality using a target trial emulation approach applied to national VA administrative data.

## 2 Methods

We conducted a retrospective cohort study to evaluate the differential impact of the SSVF program on VA healthcare utilization and costs among rural and urban Veterans experiencing housing instability. This analysis used national administrative data from VA fiscal years 2019 through 2023 (October 1, 2018 to September 30, 2023) obtained from the Veterans Health Administration Corporate Data Warehouse (CDW), which includes comprehensive information on patient demographics, housing indicators, healthcare utilization and costs within the VA healthcare system. Program enrollment and service records for SSVF participants were obtained from the Homeless Management Information System (HMIS), which tracks SSVF services delivered by grantees [17]. This study was reviewed and approved by the University of Utah Institutional Review Board and the VA Salt Lake City Research and Development Committee and is reported in accordance with the Strengthening the Reporting of Observational Studies in Epidemiology (STROBE) guidelines.

To approximate the design of a randomized controlled trial, we applied a target trial emulation framework [18] to construct a cohort of Veterans who were either enrolled in SSVF or eligible but not enrolled. A Veteran's index date, defined as the date of cohort entry, was the first documented indication of homelessness or risk of housing loss identified using ICD‐10 codes recorded in the CDW (Z59.0 for homelessness; Z59.1, Z59.8, and Z59.9 for housing instability). Eligibility criteria included being 18 years or older, having at least 1 year of prior VA healthcare records before the index date, experiencing housing instability during the study period, and having no prior SSVF enrollment. Veterans meeting eligibility criteria were classified as SSVF participants if they enrolled in the program and as eligible non‐participants otherwise. Veterans could contribute more than one eligible cohort entries prior to SSVF enrollment under the target trial emulation framework.

We estimated inverse probability of treatment weights using a propensity score model to balance baseline characteristics between the treatment and comparison groups [19]. The model included demographic (age, sex, race, and ethnicity) and clinical variables (mortality, mental health, and substance use status), participation in other VA housing programs, and lagged healthcare utilization and costs during the fourth quarter and the quarter immediately preceding the index date. These two separate lagged measures were included to capture both longer‐term baseline utilization patterns and more recent healthcare use immediately prior to cohort entry, which served as proxies for baseline health status and utilization. Mental health conditions and substance use disorder (SUD) were identified using ICD‐10 diagnostic codes recorded during the prior year. Mental health conditions were categorized into severe mental illness (e.g., schizophrenia spectrum and bipolar disorders) and other mental health disorders (e.g., depression, anxiety, and PTSD). SUD included diagnoses related to alcohol and drug use disorders.

Other VA homelessness programs included the Grant and Per Diem (GPD) program, which provides transitional housing with supportive services, and the Housing and Urban Development—Veterans Affairs Supportive Housing (HUD‐VASH) program, which combines housing vouchers with case management for permanent supportive housing. Additional programs included Domiciliary Care for Homeless Veterans (DCHV), Health Care for Homeless Veterans (HCHV) residential services, such as Contact Emergency Residential Services (CERS) and Low Demand Safe Haven (LDSH), and Compensated Work Therapy /Transitional Residence (CWT/TR). Participation in these programs was included as a covariate because Veterans may engage with multiple VA housing services over time [20]. These weights generated a pseudo‐population in which measured baseline covariates were balanced across groups, allowing estimation of intervention effects without further covariate adjustment [21, 22]. To reduce the influence of extreme weights, inverse probability weights were trimmed at the 99th percentile of the weight distribution [23].

Rurality was defined using geographic information from the VA electronic health record and enrollment files. Veterans’ residential locations were classified as highly rural, rural, or urban using Rural–Urban Commuting Area (RUCA) codes [24] applied to two Veterans’ address sources: (1) the address linked to an outpatient encounter in the CDW, and (2) the most recent address recorded in the Planning Systems Support Group (PSSG) Enrollee File prior to the index date. The CDW address was used when available, and the PSSG address was used to supplement missing CDW address information. Agreement between the two sources was 83%, and combining both sources reduced missing rurality classification from 1.23% to 0.2%. Veterans classified as highly rural based on RUCA codes were grouped with rural Veterans due to the small size of this subgroup.

Primary outcomes included quarterly VA healthcare utilization and costs, measured over a 12‐quarter observation window (four pre‐index and eight post‐index quarters). Utilization outcomes included total inpatient length of stay (LOS; days per quarter), number of outpatient visits, and number of emergency department visits per quarter. Cost outcomes included inpatient, outpatient, and ED costs, adjusted to 2023 US dollars using the Personal Consumption Expenditures (PCE) index [25].

To estimate the effects of SSVF enrollment on utilization and costs, we employed generalized estimating equations with appropriate distributions for each outcome. A negative binomial distribution with a log link was used for count‐based outcomes, including inpatient length of stay, outpatient visits, and emergency department visits, while a Poisson or a gamma distribution with a log link was applied for cost outcomes. Each model included interaction terms between treatment status (SSVF enrollment) and time (quarter) to estimate post‐enrollment changes, and three‐way interactions among treatment status, time, and rurality to evaluate whether the effects of SSVF differed between rural and urban Veterans. This specification represented a difference‐in‐difference‐in‐differences (DDD) framework that formally tested the statistical significance of rural–urban differences in SSVF effects over time [26]. All models incorporated the inverse probability of treatment weights derived from the propensity score model to adjust for baseline differences between groups, used an exchangeable working correlation structure to account for within‐individual correlation across quarters, and applied robust standard errors for repeated measures. Analyses were conducted using SAS version 9.4 [27] and Stata version 18 [28].

## 3 Results

### 3.1 Descriptive Characteristics

The analytic sample included 585,456 eligible cohort entries contributed by 171,079 unique Veterans, and approximately 16.3% of the study cohort resided in rural areas, with similar proportions in the SSVF and non‐SSVF Veterans. Table 1 presents descriptive statistics comparing Veterans with and without SSVF participation, stratified by rural versus urban residence. Rural and urban Veterans were similar in mean age 57 ± 14 years and sex distribution (about 9% female). Rural Veterans were more likely to be White (66% vs. 49% among urban) and non‐Hispanic (89% vs. 86%). Participation in other VA homelessness programs during the prior year was less common among rural Veterans, particularly GPD (3% vs. 4%) and HUD‐VASH enrollment (22% vs. 33%). Mental health and SUD diagnoses during the prior year were highly prevalent in both groups, with any mental health condition affecting roughly two‐thirds of Veterans (>60%), and any SUD recorded in about 44%. Mortality rates were similar across Veteran cohort (4–5%). Within both rural and urban settings, Veterans were mostly similar across SSVF and non‐SSVF groups in terms of age, sex, and racial/ethnic composition. The primary differences were that non‐SSVF Veterans were more likely to have a prior‐year mental health or SUD diagnosis and less likely to be enrolled in HUD‐VASH than SSVF participants.

**TABLE 1:** Descriptive statistics on Veterans living in rural vs. urban areas.

	Rural				Urban			
	Non‐SSVF		SSVF		Non‐SSVF		SSVF	
	*N* = 91,034		*N* = 4334		*N* = 466,375		*N* = 22,694	
	*N*	%	*N*	%	*N*	%	*N*	%
Age, mean (SD)	56	14	56	14	57	14	56	14
Sex								
Female	8249	9%	451	10%	41,511	9%	2341	10%
Race								
White	59,738	66%	2865	66%	230,369	49%	10,946	48%
Black	19,454	21%	909	21%	177,654	38%	8683	38%
Asian/Pacific Islander/Native Americans	4306	5%	208	5%	17,014	4%	915	4%
Missing/unknown	7536	8%	352	8%	41,338	9%	2150	9%
Ethnicity								
Non‐Hispanic	80,783	89%	3843	89%	400,064	86%	19,284	85%
Hispanic	3892	4%	214	5%	35,092	8%	1722	8%
Declined/unknown	6359	7%	277	6%	31,219	7%	1688	7%
Mortality								
Died	4997	5%	211	5%	24,567	5%	975	4%
Mental health/substance use status (Pre 1 year)								
Any severe mental health condition	16,393	18%	637	15%	92,484	20%	3411	15%
Any mental health condition	58,421	64%	2665	61%	291,959	63%	13,445	59%
Any SUD	39,277	43%	1658	38%	205,266	44%	8839	39%
VA Homelessness Program enrollment status (Pre 1 year)								
GPD	2771	3%	88	2%	19,823	4%	751	3%
HUD‐VASH	19,209	21%	1669	39%	148,306	32%	10,960	48 %
Other programs	5924	7%	257	6%	30,379	7%	1292	6%

SSVF, Supportive Services for Veteran Families; SUD, substance use disorder; GPD, Grant and Per Diem Program; HUD‐VASH, US Department of Housing and Urban Development‐Veterans Affairs Supportive Housing.

*Note*: Other programs include Domiciliary Care for Homeless Veterans (DCHV), Health Care for Homeless Veterans (HCHV) residential services, such as Contact Emergency Residential Services (CERS) and Low Demand Safe Haven (LDSH), and Compensated Work Therapy /Transitional Residence (CWT/TR).

### 3.2 Descriptive Unadjusted Utilization and Costs

Table 2 presents unadjusted VA utilization and costs for the year before SSVF index and the first and second post‐index years, stratified by SSVF enrollment and rurality. Across settings, SSVF participants in both urban and rural areas showed declines in inpatient days from the pre‐index year to the first post‐index year (urban: 16 to 8 days; rural: 15 to 9 days), with levels remaining lower in the second post‐index year (both 6 days). Outpatient visits increased during the first post‐index year among SSVF participants (urban: 32 to 43 visits; rural: 26 to 41 visits) and then stabilized or declined slightly by year 2. Emergency department visits were comparatively low in all groups and changed little over time.

**TABLE 2:** Unadjusted yearly mean utilization and costs for Veterans living in urban and rural areas: stratified by SSVF enrollment.

		Non‐SSVF			SSVF		
		Pre 1 year	Post 1 year	Post 2 years	Pre 1 year	Post 1 year	Post 2 years
**Utilization; mean (SD)**							
**Urban**	Inpatient	20 (54)	16 (50)	9 (36)	16 (49)	8 (28)	6 (26)
	Outpatient	37 (34)	39 (36)	28 (31)	32 (32)	43 (37)	28 (31)
	ED	1.8 (4)	1.5 (3.8)	1.2 (3.3)	1.4 (2.9)	1.2 (2.6)	1 (2.4)
							
**Rural**	Inpatient	21 (52)	18 (52)	10 (37)	15 (42)	9 (29)	6 (24)
	Outpatient	31 (29)	34 (33)	24 (29)	26 (26)	41 (35)	26 (30)
	ED	1.3 (2.8)	1.1 (2.7)	0.9 (2.4)	1.1 (2.3)	1.1 (2.7)	0.8 (2.3)
**Costs; mean (SD)**							
**Urban**	Inpatient	$37,260 ($96,447)	$32,615 ($101,318)	$22,420 ($85,082)	$28,683 ($76,045)	$17,649 ($60,409)	$15,616 ($65,772)
	Outpatient	$22,920 ($23,855)	$24,754 ($26,096)	$17,869 ($22,983)	$19,658 ($21,530)	$27,385 ($26,084)	$18,393 ($22,563)
	ED	$2546 ($6436)	$2230 ($6344)	$1727 ($5657)	$1989 ($5465)	$1747 ($4336)	$1417 ($3997)
	Total	$60,180 ($101,450)	$57,368 ($106,438)	$40,288 ($90,914)	$48,341 ($81,638)	$45,034 ($69,640)	$34,009 ($73,137)
							
**Rural**	Inpatient	$38,658 ($94,068)	$33,757 ($99,260)	$21,600 ($79,713)	$26,894 ($73,328)	$18,464 ($57,964)	$13,976 ($52,017)
	Outpatient	$17,607 ($19,046)	$20,335 ($22,747)	$14,716 ($19,715)	$15,107 ($16,742)	$24,935 ($24,177)	$16,168 ($20,914)
	ED	$1842 ($4147)	$1604 ($4156)	$1247 ($3951)	$1449 ($3503)	$1519 ($4087)	$1160 ($3488)
	Total	$56,265 ($98,562)	$54,092 ($103,916)	$36,316 ($84,973)	$42,001 ($77,655)	$43,399 ($66,711)	$30,144 ($60,789)

ED: emergency department; SD: standard deviation; SSVF: Supportive Services for Veteran Families.

The corresponding cost patterns followed similar trends. Among SSVF participants, inpatient costs decreased from the pre‐index year to the first post‐index year (urban: $28,683 to $17,649; rural: $26,894 to $18,464) and remained below pre‐index levels in the second year. Outpatient costs increased during the first year (urban: $19,658 to $27,385; rural: $15,107 to $24,935) and were largely unchanged thereafter, while ED costs remained relatively small and changed little over time. Among eligible non‐SSVF Veterans, year‐to‐year patterns were flatter, with smaller changes in inpatient and outpatient utilization and costs. These unadjusted summaries indicate that within VA care, SSVF participation was associated with lower inpatient intensity and modest increases in outpatient use, although rural–urban differences were not consistent enough in the descriptive data alone to suggest stronger effects in one setting versus the other.

### 3.3 Adjusted Quarterly Inpatient Length of Stay and Cost

**FIGURE 1:**
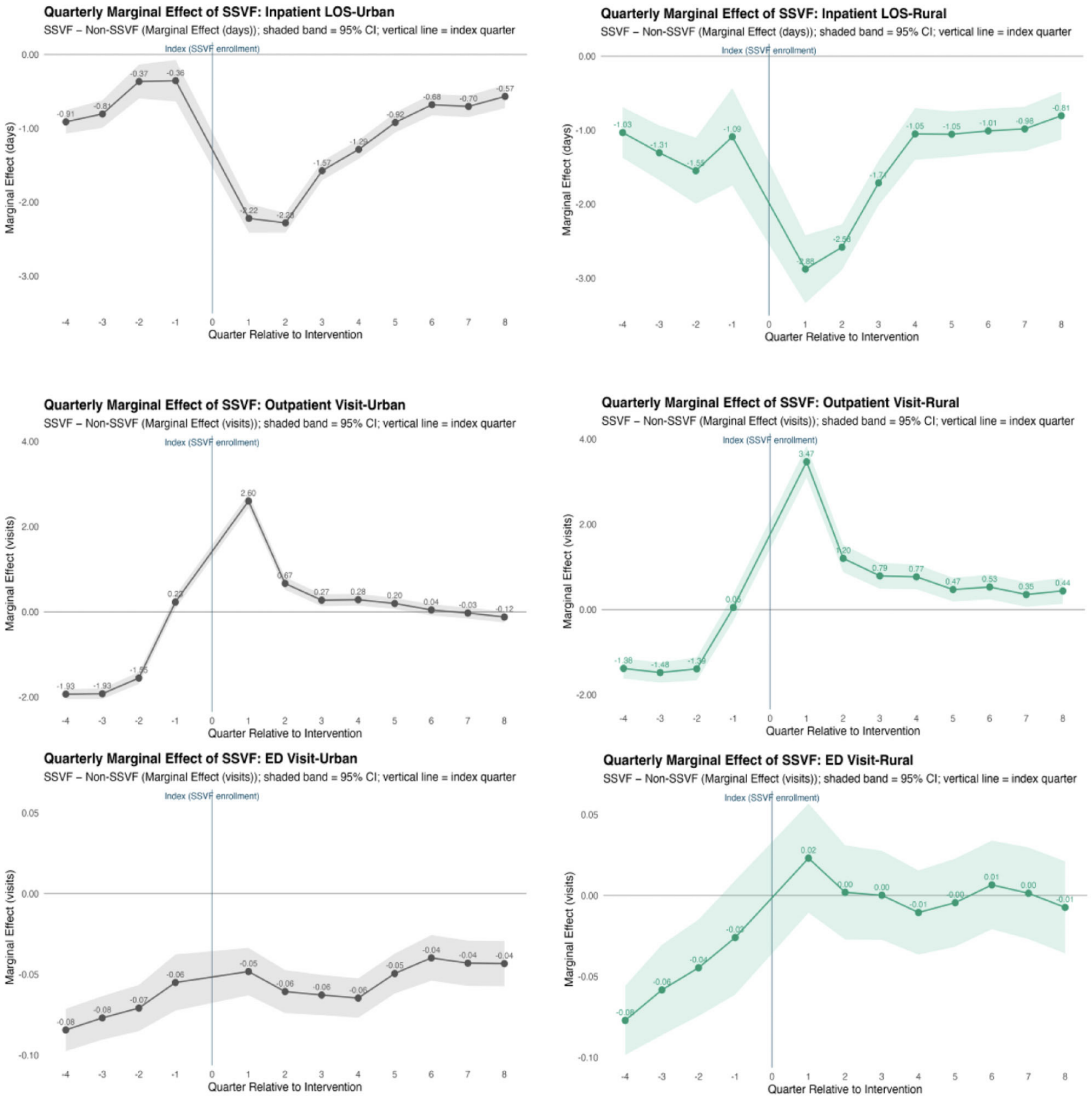
Quarterly adjusted differences associated with SSVF enrollment, stratified by rural vs. urban: inpatient days, number of outpatient and ED visits (GEE with negative binomial and log link). *Note*: Shaded areas represent 95% confidence intervals. Estimates are derived from a generalized estimating equations (GEE) with negative binomial distribution and log link, including an interaction term between quarter and SSVF. LOS, length of stay; SSVF, Supportive Services for Veteran Families.

Figure 1 shows that SSVF participation was associated with substantial reductions in adjusted inpatient LOS among both urban and rural Veterans. In the early post‐index period, inpatient LOS decreased by about 2.2 days for urban Veterans and nearly 3.0 days for rural Veterans per quarter, relative to eligible non‐SSVF Veterans. After this initial decline, the effect stabilized, with average hospital stays remaining roughly 0.6–0.7 days shorter for urban Veterans and about 1.0 day shorter for rural Veterans during the remainder of follow‐up (Figure 1).

**FIGURE 2:**
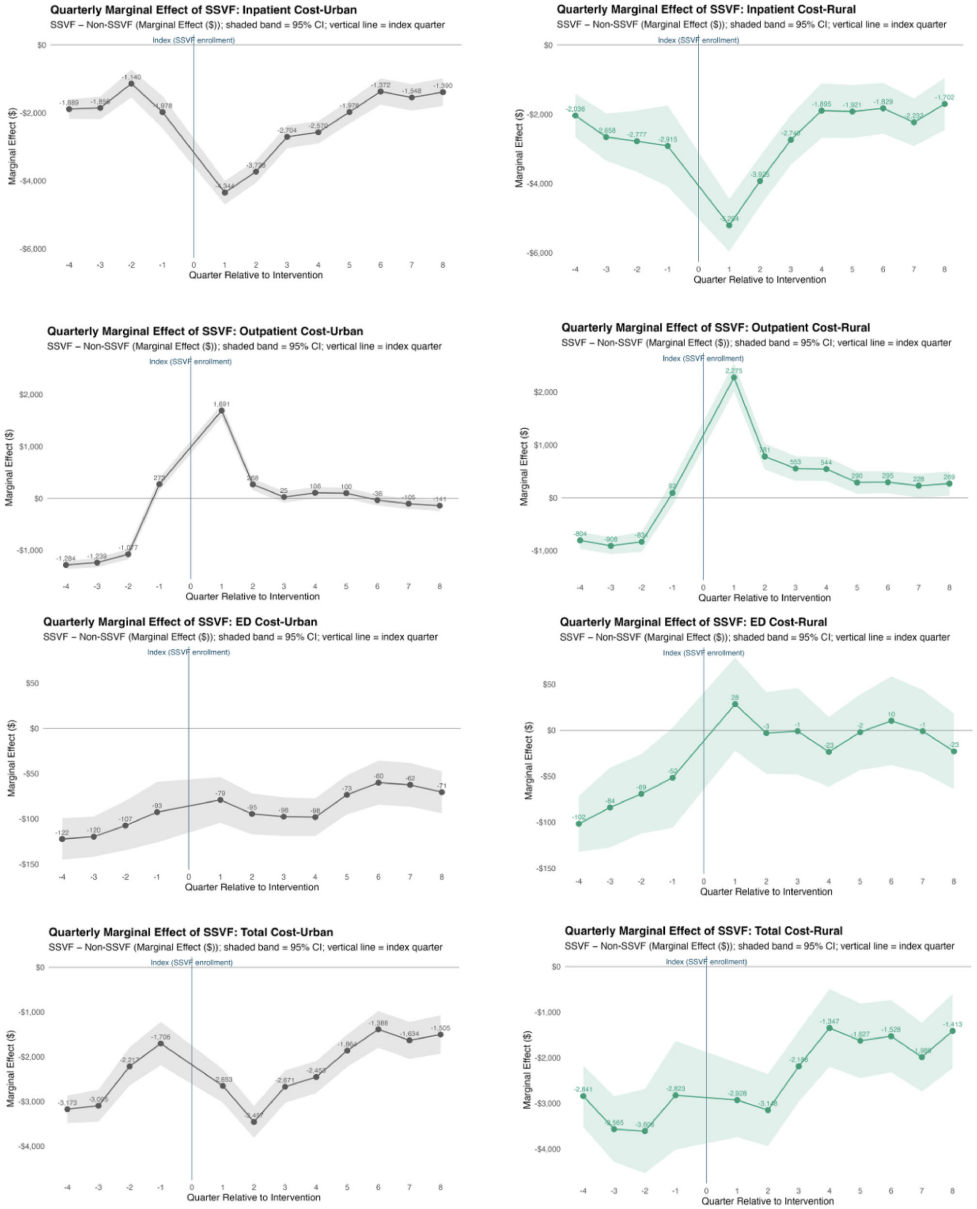
Quarterly adjusted differences associated with SSVF enrollment, stratified by rural vs. urban: inpatient, outpatient, ED and overall cost (GEE with gamma function and log link) *Note*: Shaded areas represent 95% confidence intervals. Estimates are derived from a generalized estimating equations (GEE) with Poisson or gamma distribution and log link, including an interaction term between quarter and SSVF. SSVF, Supportive Services for Veteran Families.

A similar pattern was observed for corresponding VA inpatient costs. During the first two post‐index quarters, inpatient costs decreased by about $4300 for urban Veterans and $5300 for rural Veterans, before partially returning toward baseline. Although the size of the reduction diminished after approximately 1 year, modest savings persisted into the second year, averaging around $1500 for urban and over $2000 for rural Veterans each quarter (Figure 2).

The triple interaction of time, SSVF participation, and rurality was not statistically significant for either inpatient LOS or inpatient cost, suggesting that while reductions appeared somewhat larger and more sustained in rural settings, these rural–urban differences were not statistically meaningful (Table A1).

### 3.4 Adjusted Quarterly Outpatient Utilization and Costs

SSVF participation was associated with increases in outpatient utilization for both urban and rural Veterans. Among urban Veterans, outpatient visits increased by 2.6 additional visits relative to non‐SSVF Veterans during the first post‐index quarter but declined thereafter, approaching nearly zero in later follow‐up quarters. Rural Veterans showed a slightly larger initial increase (about 3.5 visits) and continued to experience modest but sustained increases in outpatient utilization, around 0.5–1.0 additional visit per quarter throughout the follow‐up period, suggesting improved engagement in outpatient care following SSVF enrollment (Figure 1).

Changes in outpatient costs mirrored changes in outpatient visits. Urban Veterans experienced an initial increase of about $1700 in the first post‐index quarter, but the effect diminished thereafter. Rural Veterans showed a larger initial cost increase of about $2300 in the first quarter and maintained modest but sustained cost differences of roughly $200‐$800 per quarter throughout the follow‐up period (Figure 2).

The triple interaction of time, SSVF participation, and rurality was not statistically significant for either outpatient visits or costs, indicating that the effects of SSVF on outpatient utilization and spending did not differ significantly between rural and urban Veterans (Table A1). Although the descriptive patterns suggest somewhat more sustained increases in outpatient engagement among rural Veterans, these differences were not statistically meaningful across settings.

### 3.5 Adjusted Quarterly Emergency Department Utilization and Costs

SSVF participation was associated with small differences in ED use among urban Veterans and no statistically significant change among rural Veterans. Among urban Veterans, the number of VA ED visits was slightly lower for SSVF participants than for non‐SSVF Veterans throughout the study period, averaging about 0.1 fewer visits per quarter. Among rural Veterans, ED utilization showed little or no measurable change following SSVF enrollment, with greater variability across quarters (Figure 1).

Patterns for ED costs were broadly consistent with ED utilization results. Among urban Veterans, ED costs for SSVF participants remained modestly lower than for non‐SSVF Veterans across both pre‐ and post‐index quarters, ranging from about $60 to $120 lower per quarter (Figure 2). In the DDD models, several quarter‐specific interaction terms between SSVF participation, quarter, and rurality were statistically significant, indicating that the estimated SSVF effects on ED utilization and costs differed between rural and urban Veterans, although the magnitude of these differences was small (Table A1).

### 3.6 Adjusted Quarterly Total VA Costs

When examining overall VA costs, SSVF participation was associated with a clear and substantial reduction during the early post‐index period, followed by gradual stabilization over time for both urban and rural Veterans. In the early post‐index period, total costs were approximately $3000 lower per quarter among SSVF participants relative to non‐SSVF Veterans. Over time, the magnitude of this difference gradually diminished but remained negative throughout follow‐up, with costs about $1400–$2000 lower per quarter in later quarters (Figure 2).

SSVF effects on total costs were similar for rural and urban Veterans. Although rural Veterans appeared to experience slightly larger cost differences in some quarters, the triple interaction of time, SSVF participation, and rurality was not statistically significant (Table A1).

## 4 Discussion

SSVF participation was associated with reductions in inpatient LOS and costs and modest increases in outpatient engagement among Veterans experiencing housing instability. In both rural and urban settings, inpatient days and corresponding inpatient costs declined significantly in the early post‐enrollment period, while outpatient visits increased modestly, suggesting greater engagement with routine and preventive care following program participation. Emergency department use showed modest reductions among urban Veterans and no measurable change among rural Veterans. These changes corresponded with overall reductions in total VA costs during the early post‐enrollment period, with costs remaining lower than those of non‐SSVF Veterans throughout follow‐up. Although point estimates were occasionally different between rural and urban Veterans, statistical tests did not indicate statistically significant rural–urban differences in the overall patterns of utilization or spending. Together, these findings suggest that SSVF participation is associated with shifts away from expensive acute care and toward greater outpatient engagement within the VA healthcare system.

These findings align with prior evaluations of SSVF and other homelessness prevention programs within the VA system. Previous studies have shown that housing support programs are also associated with reductions in inpatient utilization and shifts toward outpatient care among Veterans experiencing housing instability [15, 16]. Nelson et al. found that SSVF's Temporary Financial Assistance was associated with decreases in inpatient costs and shifts in health care spending toward less intensive care settings [15]. More recent national analyses of homelessness prevention services similarly reported declines in acute care use following program participation [16]. This study extends this literature by carefully comparing rural and urban Veterans to examine whether the health care effects of housing stabilization differ across geographic settings. Our results suggest that the benefits of SSVF are broadly shared across settings and do not differ based on rural–urban status, with similar patterns of reduced inpatient utilization and increased outpatient engagement among both rural and urban Veterans.

This study implies the potential role of housing stabilization programs in shaping healthcare utilization patterns within the VA healthcare system. By helping Veterans secure stable housing, SSVF may reduce reliance on costly acute care and support greater engagement with routine outpatient care. These patterns were observed among both rural and urban Veterans, indicating that the health care benefits associated with SSVF participation are not limited to urban settings where VA services are more concentrated. Ensuring that housing stabilization programs remain well integrated with VA healthcare services may help sustain these changes in care patterns and support continuity of care for Veterans experiencing housing instability across diverse geographic settings.

This study has several limitations. First, the analyses capture only VA‐provided care and therefore do not reflect utilization or costs that occurred through community care programs or non‐VA providers. This limitation is particularly relevant for rural Veterans, who are more likely to rely on non‐VA services [29, 30]. Second, although inverse probability weighting reduced baseline imbalances between groups, unmeasured differences in clinical need, housing instability severity, or motivation to seek services may remain. For example, Veterans who enroll in SSVF may differ from eligible non‐participants in unobserved characteristics such as severity of housing instability, engagement with social services, health status not captured in claims data, or referral patterns from VA providers, which also could influence follow‐up health care utilization. Third, administrative data may incompletely capture social determinants of health. ICD codes used to document social determinants, including homelessness and housing instability, are not consistently recorded in clinical documentation [31, 32]. Therefore, some Veterans experiencing housing instability may not have been identified in the administrative data used to define the study cohort. Finally, the study period of eight post‐index quarters allows for evaluation of medium‐term effects but does not capture longer‐term effects of cost and utilization.

Future research should further examine how housing stabilization programs such as SSVF are accessed and implemented in rural communities to better understand potential differences in program reach and participation. Although this study found broadly similar healthcare effects among rural and urban Veterans who participated in SSVF, it remains unclear whether rural Veterans are able to enroll in and benefit from the program in the same way as urban Veterans. Understanding barriers and facilitators to program participation, including through qualitative or mixed‐methods research, will also be important for ensuring that the benefits of housing stabilization programs reach Veterans across geographic settings. Future work that incorporates both VA and non‐VA healthcare utilization would also provide a more complete picture of overall healthcare use, particularly for rural Veterans who receive substantial care outside the VA system.

## 5 Conclusion

SSVF participation was associated with reduced inpatient utilization and costs and modest increases in outpatient engagement among Veterans experiencing housing instability. These effects were observed among both rural and urban Veterans, suggesting that the healthcare benefits of housing stabilization are present in both urban and rural populations within the VA system. Continued efforts to maintain and strengthen housing stabilization programs may therefore contribute to improved health care utilization patterns and stability for Veterans experiencing housing instability.

## Funding

This work was supported by the US Department of Veterans Affairs, Office of Rural Health (ORH; PROJFY‐010205). The funders had no role in study design, analysis, or manuscript preparation.

## Ethics Statement

This study was approved by the University of Utah Institutional Review Board and the VA Salt Lake City Research and Development Committee through an expedited review process (45 CFR 46.110), with a waiver of authorization for retrospective data use.

## Conflicts of Interest

The authors declare no competing interests related to this study.

## 1

**TABLE A1:** Difference‐in‐difference‐in‐differences (DDD) estimates of SSVF effects on VA utilization and costs by rurality.

	Utilization			Cost		
**Inpatient visit**						
Quarter relative to enrollment	DDD coefficient	95% CI	*p*‐value	DDD coefficient	95% CI	*p*‐value
−3	−0.13	(–0.29, 0.02)	0.098	−0.13	(–0.3, 0.04)	0.148
−2	−0.20	(–0.4, –0.01)	0.036	−0.13	(–0.33, 0.06)	0.181
−1	0.00	(–0.19, 0.18)	0.989	0.04	(–0.16, 0.23)	0.697
+1	0.03	(–0.17, 0.22)	0.802	0.03	(–0.17, 0.24)	0.742
+2	0.00	(–0.24, 0.24)	0.990	0.03	(–0.21, 0.27)	0.797
+3	0.06	(–0.18, 0.3)	0.627	0.06	(–0.18, 0.3)	0.601
+4	0.25	(0.01, 0.49)	0.042	0.21	(–0.03, 0.46)	0.080
+5	0.04	(–0.21, 0.28)	0.771	0.08	(–0.17, 0.33)	0.552
+6	−0.09	(–0.34, 0.16)	0.495	−0.04	(–0.29, 0.21)	0.768
+7	−0.07	(–0.32, 0.18)	0.600	−0.10	(–0.35, 0.15)	0.428
+8	−0.05	(–0.3, 0.21)	0.728	−0.01	(–0.27, 0.24)	0.913
**Outpatient visit**						
Quarter relative to enrollment	DDD coefficient	95% CI	*p*‐value	DDD coefficient	95% CI	*p*‐value
−3	−0.03	(–0.08, 0.01)	0.169	−0.07	(–0.14, 0)	0.046
−2	−0.07	(–0.13, –0.01)	0.014	−0.08	(–0.16, 0)	0.039
−1	−0.06	(–0.12, 0)	0.037	−0.09	(–0.17, –0.02)	0.016
+1	0.04	(–0.02, 0.1)	0.173	0.04	(–0.04, 0.11)	0.335
+2	0.02	(–0.04, 0.08)	0.595	0.03	(–0.05, 0.1)	0.534
+3	0.02	(–0.05, 0.08)	0.597	0.03	(–0.05, 0.11)	0.449
+4	0.02	(–0.05, 0.08)	0.602	0.02	(–0.06, 0.1)	0.626
+5	−0.01	(–0.07, 0.05)	0.806	−0.03	(–0.11, 0.05)	0.510
+6	0.02	(–0.04, 0.08)	0.508	0.00	(–0.08, 0.08)	0.953
+7	0.01	(–0.06, 0.07)	0.805	0.00	(–0.08, 0.09)	0.939
+8	0.04	(–0.03, 0.1)	0.299	0.02	(–0.06, 0.11)	0.604
**ED visit**						
Quarter relative to enrollment	DDD coefficient	95% CI	*p*‐value	DDD coefficient	95% CI	*p*‐value
−3	0.08	(–0.08, 0.23)	0.329	0.07	(–0.11, 0.24)	0.448
−2	0.11	(–0.03, 0.26)	0.129	0.09	(–0.07, 0.24)	0.283
−1	0.13	(0, 0.26)	0.042	0.11	(–0.04, 0.25)	0.143
+1	0.27	(0.14, 0.41)	<0.001	0.26	(0.11, 0.42)	0.001
+2	0.27	(0.12, 0.41)	<0.001	0.25	(0.08, 0.41)	0.004
+3	0.28	(0.13, 0.42)	<0.001	0.27	(0.1, 0.44)	0.002
+4	0.25	(0.1, 0.4)	0.001	0.21	(0.05, 0.38)	0.012
+5	0.23	(0.07, 0.38)	0.004	0.22	(0.05, 0.39)	0.010
+6	0.24	(0.08, 0.4)	0.003	0.23	(0.04, 0.42)	0.016
+7	0.23	(0.07, 0.4)	0.005	0.20	(0.02, 0.39)	0.031
+8	0.20	(0.03, 0.37)	0.021	0.16	(–0.02, 0.34)	0.090
**Total cost**						
Quarter relative to enrollment				DDD coefficient	95% CI	*p*‐value
−3				−0.11	(–0.22, 0)	0.056
−2				−0.12	(–0.25, 0.01)	0.064
−1				−0.03	(–0.15, 0.1)	0.667
+1				0.01	(–0.12, 0.13)	0.930
+2				0.03	(–0.11, 0.16)	0.707
+3				0.05	(–0.09, 0.19)	0.494
+4				0.11	(–0.03, 0.25)	0.111
+5				0.03	(–0.12, 0.17)	0.708
+6				−0.01	(–0.16, 0.14)	0.884
+7				−0.04	(–0.19, 0.11)	0.582
+8				0.01	(–0.14, 0.16)	0.882

*Note*: Estimates are derived from generalized estimating equation models with a log link and exchangeable correlation structure. Utilization outcomes were modeled using negative binomial distributions, and cost outcomes were modeled using Poisson or gamma distributions. Models incorporated inverse probability weights and robust standard errors clustered at the patient‐trial level. Coefficients represent quarter‐specific triple interaction terms (SSVF × quarter × rural).

SSVF, supportive Services for Veteran Families; CI, confidential Intervals.
